# Repeat testing for chlamydia trachomatis, a “safe approach” to unsafe sex? a qualitative exploration among youth in Stockholm

**DOI:** 10.1186/s12913-017-2681-6

**Published:** 2017-11-15

**Authors:** Anna Nielsen, Ayesha De Costa, Kristina Gemzell Danielsson, Mariano Salazar

**Affiliations:** 1 Department of Women′s and Children′s Health K6, Karolinska University Hospital Solna, 17176 Stockholm, Sweden; 2 Department of Public Health Sciences, Karolinska Institutet, Tomtebodavägen 18 A, 171 77 Stockholm, Sweden

**Keywords:** Chlamydia trachomatis, Youth, Repeat testing, Stigma, Sexual health, Sweden, Sexual behaviour, Health care utilization

## Abstract

**Background:**

*Chlamydia trachomatis* testing is offered to youth in Sweden, through a network of Youth Health Clinics, free at the point of care, in an attempt to bring down the prevalence and incidence of the infection. Nevertheless, infections rates have continued to rise during the past two decades and re-testing rates among youth for *Chlamydia trachomatis* has been reported to be high in Stockholm County. A few literature reports suggest that testing for sexually transmitted infections and the test result itself can have an undesirable impact on the sexual behaviour for the individual, i.e. increase sexual risk-taking.

**Methods:**

This qualitative study aimed to explore the motives for repeated testing for *Chlamydia trachomatis* among youth using the services of the Youth Health Clinics in Stockholm, and how testing affects their subsequent risk-taking. We interviewed 15 repeat testers aging 18–22 years.

**Results:**

Our main findings were that the fear of social stigma related to infecting a peer was a major driver of the re-testing process. The repetitive testing process, the test result, and the encounter with personnel did not decrease sexual risk-taking among this group.

**Conclusions:**

While testing and treatment services are an important part of *Chlamydia trachomatis* prevention it must not take the focus away from primary prevention strategies. Testing should be encouraged, but not to the exclusion of risk reduction measures. The testing services must be complemented with stronger emphasis on safe sex, especially for those who attend the clinics repeatedly, otherwise the easy accessible testing services risk counteracting its own purpose. Future research should focus on developing and evaluating youth appropriate interventions to increase condom use, taking into consideration factors which youth perceive as important to drive this behaviour change.

**Electronic supplementary material:**

The online version of this article (10.1186/s12913-017-2681-6) contains supplementary material, which is available to authorized users.

## Background

*Chlamydia trachomatis (C.trachomatis)* is the most commonly reported sexually transmitted infection (STI) in Sweden and worldwide, most commonly affecting young adults [1, 2]. In an attempt to control the epidemic, several countries have introduced population-based screening programs or opportunistic screening programs [1]. In Sweden, an opportunistic screening approach, which began in the 1980s, is available across the country [3].

The most effective way to tackle the epidemic of *C.trachomatis* is still being debated, and different guidelines on *C.trachomatis* screening exist in different settings [4]. For example, in the United States re-testing for *C.trachomatis* 3 months after diagnosis is recommended, [5]. In the United Kingdom, the National *C.trachomatis* Screening Program aims to test people under the age of 25 once a year [6].

The Swedish opportunistic screening program has been held up as a model program [7]. Approximately 500,000 *C.trachomatis* tests are performed in the country per year (2011–2015) with an overall positivity rate of six to 7 % [2]. Women account for 57% of all reported cases. The median age for infected individuals is 22 years for women and 24 years for men [2]. Guided by national laws and regulations [8, 9], regional policies control the preventive work in the different County Councils in Sweden [10]. Repeat testing after an infection is not official policy in Stockholm County [10].

Nevertheless, repeated testing for *C.trachomatis* is relatively common behaviour among youth living in Stockholm County. In a recent study, we found that 42% of youth using the testing services at the Youth Health Clinics (YHC) in Stockholm re-tested for *C.trachomatis* during a 3 year period [11]. The repeat testers in our study tested between two to 19 times over the study period [11]. Re-testing was associated with being female, a previous positive test and living in a high- or middle-income area of Stockholm. Furthermore, we found high rates of *C.trachomatis* among repeat testers both at baseline and at repeat tests which suggests the possibility that this group might be continuing to engage in unsafe sexual practices [11]. However, the reasons for repeat *C.trachomatis* testing on an individual level are still unknown.

There is some evidence in the literature which suggests that a positive test result for a STI might affect sexual risk taking promoting a more protective disposition, at least in the short term [12, 13]. A negative STI test result, on the other hand, seems to have no effect on subsequent risk taking [12]. Studies focusing on HIV testing indicate that a negative test result can have an undesirable effect on behavioral changes, i.e. adopting more risky sexual practices [14–17].

Re-testing for *C. trachomatis* can have positive consequences for people and the society; i.e. positive cases are detected and treated. Thus, comprehensive testing services are therefore important. However, re-testing can also have negative consequences for health systems and individuals. For individuals, repeated testing might be a consequence of continuous risky sexual behaviour that exposes them not only to *C.trachomatis* infections but to other more serious infections such as gonorrhoea, HPV, syphilis, and HIV.

For health systems, repeated testing without long term behavioural change might not reduce *C.trachomatis* incidence and therefore can result in an inefficient use of resources. In addition, repeated infections and subsequent antibiotic use contributes to increase antibiotic resistance in this population [18]. Evidence from Sweden has shown that *C.trachomatis* testing services are repeatedly used by a proportion young adults [11], who, in spite of their constant exposure to testing services, continue to exhibit risky sexual behaviours. This, together with the reported high rates of *C.trachomatis* in Stockholm County [2],might indicate that health care resources have not been effectively used to reduce the incidence of *C.trachomatis* infections in this setting.

### Objectives and rationale

In order to strengthen the opportunistic screening program in Sweden it is important to explore the reasons behind the high *C.trachomatis* re-testing figures previously reported in this setting [11]. Thus, this qualitative study aims to explore the motives for repeat testing for *C.trachomatis* among youth using the services of the Youth Health Clinics in Stockholm, and how testing affects their sexual risk-taking. The results of this study will allow the *C.trachomatis* screening program to design and implement appropriate interventions to work with youth retesting frequently and take steps make the program more effective. In addition, the lessons learned from our study might be helpful to other screening programs dealing with high re-testing rates.

### Theoretical framework

We have chosen the Andersen’s Model of Health Service Utilization as theoretical framework for this study [19]. In the model, usage of health services is determined by three dynamics: *predisposing factors, enabling factors, and need.* Predisposing factors include characteristics such as gender, age, ethnicity, and health beliefs. Enabling factors include family support, income, and accessibility to health care in the community. Need represents both perceived and actual need for health care services [20].

The model used in this paper was adapted from Andersen in 1995; the phase four model [20]. This model describes a number of interacting factors influencing health service utilization. These factors are: *1. The Environment* (health care system and external environment such as physical, political and economic environment), *2. Population characteristics* as mentioned above (predisposing factors, enabling factors and need). *3. Health Behaviour* (personal health practices and use of the health care system) and *4. Outcome* (perceived health status, evaluated health status and customer satisfaction). The model also recognizes that outcome and consumer satisfaction affects the *predisposing factors*, the perceived *need* and the *use* of health services [20].

## Methods

### Setting

The study was conducted at the YHC in Stockholm County, Sweden. Stockholm County is spread over an area of 6488 km^2^, and contains both urban and rural areas. The total population is approximately 2.2 million and youth (15–24 year old) account for approximately 11.6% of the population [21].

Stockholm has one of the highest *C.trachomatis* infection rates in the country with 459 cases/100,000 inhabitants in 2015 [2]. In 2015, 10,144 cases of *C.trachomatis* were identified in the county representing a 7 % increase from the previous year. Most cases were found in the age group 20–24 (37%) years and more than half (54%) were in women [2]. Approximately 40% (2012) of all positive tests in Stockholm were carried out at the YHC [22].

### Youth health clinics

Sweden is a high income country with universal health care provided by the state [23]. Health care for young people is free at the YHC. In 1970 the first YHC was launched in Sweden. Sexual health and development was and is the main focus of the YHC [24]. The staff comprise of nurse/midwives, behavioural therapists, social workers and physicians. In the whole country there are approximately 220 clinics with 33 located in Stockholm County [25, 26].

The target age for the YHC ranges from 12 to 23 years old. The clinics are involved both in primary prevention of STI, (i.e. sexual education to school classes, counselling of clients and condom information/distribution), and secondary prevention (i.e. testing and treatment services). Opportunistic screening is incorporated into its services. In 2014, 113,000 visits were made by approximately 56,000 unique visitors to the YHC in Stockholm [26].Approximately 40,000 *C.trachomatis* tests are performed in the YHC in Stockholm each year [11].

### Participant selection

Participants were recruited using a non-probabilistic stratified sample [27]. Respondents for interviews (youth who came to the clinic) were selected purposefully, so that both sexes and persons from different socio-economic zones of Stockholm were represented in the sample.

The participants were initially approached when attending the YHC for a *C.trachomatis* test. A person who was identified as a repeat tester (tested more than once during a 6 month period) by the clinic midwife was informed briefly about the study. If the young person showed interest in participating in the study they were asked for permission to be contacted by a member of the research team for more detailed information. If such permission was given, then the young person was contacted by the first author (AN), who provided oral and written information about the purpose of the study, answered any questions or clarifications and invited the young person to participate in an in-depth interview.

In total we gathered data from 15 in-depth interviews (eight female and seven male) from eight different YHC situated in different areas of Stockholm (inner city and suburbs). Saturation was reached after 15 interviews. The age of the participants ranged from 18 to 22 years. All participants were unmarried and no one was currently in a steady relationship. All participants except one had finished upper secondary school. Most interviewees were living with their family (9/15). Equally many participants were studying at university as those employed. Three participants were looking for a job. In all interviews the participants referred to partners of the opposite sex while discussing sexual practices.

### Data collection

The in-depth interviews were conducted at a quiet location chosen by the participant. A trained qualitative researcher, the first author (AN) conducted all interviews, which took from 33 to 55 min. The interviews were conducted in Swedish and were recorded using a mobile phone device. The recordings where sent to a computer via e-mail, downloaded and saved on a password protected computer. The interviews were transcribed verbatim in Swedish. The interviews were conducted between April 2015 and August 2016.

### Interview guide

A semi-structured interview guide with open ended questions was used to gather the data (Additional file 1). Aspects that were explored included: youth perception of visiting the clinic; their experiences testing and re-testing for *C.trachomatis*, their concepts of a safe/unsafe sexual behavior and their perceptions of the consequences of being infected with *C.trachomatis.*

Follow-up, probing, and interpretive questions were used to further explore and clarify topics that were identified during the interview [28, 29]. In order to expand our understanding of the phenomenon under study, new topics that arose from the previous interviews where included in the subsequent interviews.

### Data analysis

We analyzed the data using a constructivist grounded theory approach as described by Charmaz [30]. The data collection and analysis were conducted in parallel. This allowed us to use an abductive analytical process to create emerging hypothesis based on the text and to explore them in-depth in the following interviews [30].

The process of analyzing the data began by first reading the interview transcripts and then conducting a line-by-line open coding of the data. Coding for actions was done as it facilitates the description of what is happening within the data. The codes identified in the different interviews were constantly compared with each other to reach a higher level of abstraction. This was done in parallel by two researchers and conceptual categories were created through a process of argumentation and consensus.

Memo-writing was done throughout the process of data collections to narrow the scope of the different categories, to record emerging ideas about the relationships between categories and to identify the core category. Finally, the categories were linked together in a theoretical model. Data was coded in English using OpenCode 3.4 a free software developed by Umeå University [31].

## Results

**Fig. 1:**
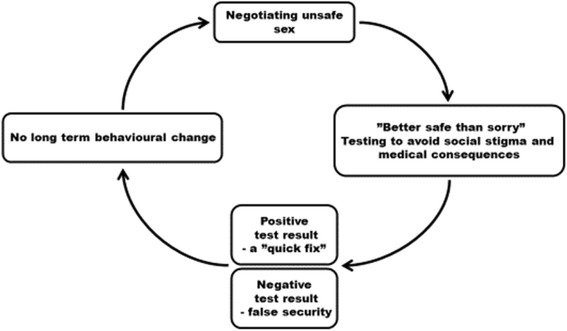
The cyclic process of testing for *C.trachomatis*

Our constructivist grounded theory analysis showed that re-testing for *C.trachomatis* is a process (Fig. 1) that contains four categories describing the different steps involved in this behavior. The categories (and their corresponding subcategories) were: *1) Negotiating unsafe sex (alcohol use, obstacles of condom use, trust and C.trachomatis not a serious infection; 2) Testing -“better safe than sorry”* (*anxiety due to social stigma related to spreading the infection and anxiety due to possible long-term medical consequences) 3) Negative test result - false security* and *4) Positive test result - a “quick fix”*. In the following we will present each category, as well as, the health care service factors facilitating the re-testing process.

### Negotiating unsafe sex

Four interacting sub-categories that promoted unprotected sex were identified. These sub-categories were; *alcohol use; obstacles to condom use; trust in sexual partner, and; C.trachomatis not a serious infection*. While negotiating unprotected sex *alcohol* stood out as an important factor increasing its likelihood and decreasing the chance of making sound decisions in the heat of the moment. Both the young men and the young women in our data talked about the *obstacles of condom use*. Sex with a condom was not considered as good as sex without a condom. Strong views on the subject were expressed;

*“I rather have no sex than have sex with a condom; I always choose pleasure before safety”* (*Interview 1, 20 year old man*).

Trusting someone to have unprotected sex with was a process where risk and rewards were constantly being evaluated. Condom use depended on how safe the other person was perceived to be, thus it became a negotiation with oneself whether to choose to trust someone and make the sexual encounter more pleasurable (without condoms) or whether to avoid unprotected sex.

The informants used several strategies to assess their potential sexual partner’s trustworthiness. For example, youth tended to ask their partner about STI status, and men additionally asked about the use of other contraceptives. In addition, trust was constructed on how well one knew a prospective sexual partner. To trust a friend or a person belonging to the same network was considered reasonably safe. However, with a casual recent acquaintance trust was built on people’s appearances including their clothing, their behaviour and how much they were attracted to each other. Conversely, it was also expressed that wanting to trust was a way of finding excuses for ones behaviour.

*“Trust is very important. At least from my perspective. Those times I had unprotected sex I like asked the girl if she had (was using) any contraceptives and it also happens that you ask about when was the last time you tested. Well, then you just trust that person’s word. And that could be someone you just dragged home after a night out. Ah, it is not so much that you can really trust in such scenario but you choose to trust just to make that moment pleasurable and because you are not used to using a condom”. (Interview 5, 21 years old man).*

Whilst negotiating risk inherent in unprotected sex, with regard to *C.trachomatis* infection it became clear that *C.trachomatis* was not considered a serious infection. *C.trachomatis* was looked upon as ‘the most preferable STI’ as it is curable in just a few days. Knowing that *C.trachomatis was* easy to cure even made youth take the risk of having unprotected sex.

*“For me it is like unprotected sex can be worth having a C.trachomatis infection. I’ll just treat with some antibiotics. Really, it can be worth it. Condom is… well it is not fun.”(Interview 2, 22 years old woman).*

Thus, negotiating risk of unprotected sex was influenced by the above mentioned interacting factors and often resulted in the decision to engage in unprotected sex. However, decisions made under the influence of alcohol and on sometimes weak grounds, such as trusting a person based on their appearances, did have consequences already on the following day. Most youth expressed that they regretted unsafe sex. Testing however assuaged their feeling of regret.

### Testing – “Better safe than sorry”

The participants expressed that repeat testing was done “just to be on the safe side”. Testing after unprotected sex was perceived to be a safe normal sexual practice and an appropriate substitute to using condoms. Testing was thought of as a way of reassuring cleanness; to see if something went wrong and to be able to do something about it. Youth expressed perceiving themselves as ‘being careful’ by testing repeatedly for *C.trachomatis*.

*“I think safe sexual practices are that you test after unprotected sex. And also maybe that you use a condom, but that’s not the most important thing. What’s important is that you test when you know you did something stupid” (Interview 4, 22 year old woman).*

As described above, anxiety was not present before, or related to the actual episode of unsafe sex. Anxiety however arose afterwards and was related to fear of long term consequences of a *C.trachomatis* infection, but foremost anxiety related to the risk of spreading *C.trachomatis* and gaining a bad reputation as a result of this.

*Testing to avoid social stigma related to a possible C.trachomatis diagnosis.*

Through the analysis it became clear that the social consequences of *C.trachomatis* were considered much more serious than the possible medical consequences. This was found to be a strong driver in the process of testing and re-testing. The aspect of shame related to infecting someone with *C.trachomatis* was identified in all interviews. Feelings of shame were present already in the waiting room of the YHC. Just being there suggested that you might have done ‘something’ and that you were most certainly there to test. To meet a friend in the waiting room was considered to increase feelings of shame and anxiety.

The discussions with the participants also revealed that infecting someone else was considered worse than being infected oneself. The shame associated with risk of spreading the disease varied depending of the level of closeness that the informant had with the sexual partner. Whereas there was no or little shame in spreading *C.trachomatis* to someone unknown, it would be very shameful to infect someone within a close circle of peers, i.e. a friend or a regular partner. This generated high levels of anxiety as the informants perceived strong negative consequences for their social life such as isolation and a “bad reputation” among their peers.

*“To infect someone is probably the most embarrassing thing you can do. People will see you as revolting. It’s like this; if a girl infects a guy he will see her as dirty. He might call her a whore. And that’s a reputation that you are stuck with.” (Interview 8, 20 year old woman).*

Another related aspect that generated anxiety was the YHC standard procedure of contacting previous sexual contacts for screening purposes. It generated anxiety because, although contact tracing is meant to be anonymous, it was considered not to be so. A general opinion was that you always could figure out who reported you to the authorities. Interestingly this anxiety could lead to increased use of condom with a friend or with someone you might want to start a relationship with. Condoms could on the other hand be ignored on a one-night stand as the social consequences of *C.trachomatis* infection were expressed to be non-existent with someone outside your own circle of friends.

### Testing to avoid long term medical consequences

A common perception among our interviewees was that having untreated *C.trachomatis* for a long time can lead to severe health consequences. However, there was a variation in their understanding of what consequences *C.trachomatis* could lead to, and an uncertainty of the duration one needed to have harboured *C.trachomatis* before such sequelae occur. It was however a prevalent opinion that the sooner one discovers *C.trachomatis* the better it is. Participants expressed that the long-term consequences can be avoided or minimized by frequent testing.

*“Before a test there is of course some worry. But I know since I’ve been so good at testing regularly, it is not the same fear. This is how I see it; if I had only tested once per year then the chance of me having something during a longer period is greater. If that was case then I would be more worried. But since I have done it regularly I am calmer. I feel like if I have it then I take my antibiotics and then I’m cured.” (Interview 6, 19 year old woman).*

### Negative test result - false security

A negative test result was seen as confirmation of the ability to choose a trustworthy “clean” sexual partner. The informants recognised that they had been careless in having sex without condoms; yet they were happy about the possibility of getting away with this risky sexual behaviour without being infected with a STI. While youth were aware that a negative test could provide a false sense of security, it did not affect any change in their risk taking behaviour. On the contrary, a negative test result made them feel invulnerable and confident, reinforcing that there was no need for a change in behaviour. Hence, negative testers continued to have unprotected sex. Repeat negative tests were even reported to make one neglect the importance of protected sex. If unprotected sex has no consequences, they believed there was no need to change behaviour.

*“Since I never got anything (an STI) then it was well, I’ll do it unprotected. I still understand that I could get something, but I have become a little foolhardy. I have become self-confident. Or maybe self-confident is not a good word but I don’t know. Stupid really, I never experienced any consequences and so it (the possibility of consequences) became more and more distant. And now I count on the test to be negative. If it would be positive I would like, oh! I do understand that it could turn out positive but I have gotten used to a negative result.” (Interview 13, 21 year old man).*

### Positive test result - “a quick fix”

A positive test was constructed as a consequence of one’s careless behaviour. It originated from several responses that ranged from identifying it as a “wake-up call”, causing some short term behavioural change, to no change at all. Within this spectrum of responses, the dominant conceptualization was that *C.trachomatis* infection *was “a quick fix*” meaning that the infection can easily be cured with a short round of antibiotics.

*“I want to say I would change my behaviour after a positive test, but I don’t know….It would not be fun if you were here for the third time and had a positive Chlamydia. But after having it once, and then you get cured pretty easy, then you would just be like…ah! I mean it’s hard to take it seriously (Chlamydia). I know it is serious, but…Do you see what I mean?” (Interview 9, 21 year old man).*

This dominant response reiterates the perceptions described in our first category where *C.trachomatis* infection was not perceived as serious as other STIs such as herpes, HPV or HIV. Consequently, a positive *C.trachomatis* test required a “quick fix” but did not merit a long term behavioural change in condom use patterns.

### Factors facilitating re-testing behaviors

The participants expressed no embarrassment testing and re-testing related to the actual test and the encounter with the staff. All interviewees had good experiences from the YHC. Feeling safe and well received at the clinic were important.

*It is very easy (testing for C.trachomatis). Super easy and super smooth. Nice to have it done, comfortable that it is for free. It is a very very easy process. (Interview 7, 19 year old woman).*

The easy accessibility to the YHC, the flexible opening hours, and the free service offered were perceived as facilitating overuse of the services. Easy access was thought to make youth take the risk of having unprotected sex. While one person said that it would not be worth having unprotected sex if the test would cost money, no one else believed that a fee of a *C.trachomatis* test would decrease sexual risk taking or increase condom use among youth. Quite the opposite was expressed; a cost would lead to fewer tests and consequently to more infections.

*You don’t want to stand there, like 2 days before pay-day and just, I really need to go and test but I don’t have the money right now. Those situations… like, you don’t have to deal with those. So I think that (free of charge) contribute a lot… that many go and test. And do it regularly. (Interview 3, 20 year old woman).*

## Discussion

Our findings show that testing repeatedly for *C.trachomatis* is a process driven by the interaction between the informants´ needs, health beliefs about *C.trachomatis* and environmental factors such as the characteristics of the Swedish Health Care System. Repeated testing was driven mainly by the informants´ fears of the social consequences (stigma, exclusion or humiliation) of spreading the disease among their close group of peers and by their conceptualization of repeated testing as a preventive measure replacing condom use. In the following, we will discuss our main findings using Andersen’s Phase four Model of Health Service Utilization [20].

### Social stigma and the need for C.Trachomatis re-testing

Anderson [20] proposes that one of the main drivers of health service utilization is the population’s *need* for specific services and this is clearly the case in our study. Our study participants expressed a strong *need* to visit the YHC as testing repeatedly would reduce anxiety related to the social consequences. While the role of stigma in relation to STI is well known, literature has mostly described stigma in relation to accepting or rejecting STI testing [32–34]. Testing and re-testing to avoid social stigma has not been previously reported.

Our finding can have important implications for *C.trachomatis* prevention. While it is important not to shame youth in relation to sexual activity and not further stigmatize STI, our results can be used to inform primary preventive strategies. Youth in our study expressed very strong feelings of regret related to unprotected sex and the anxiety of spreading the infection. Hence, strengthening youth in their decision to use a condom in relation to protect self-respect and reduce anxiety is a way forward. As previously been reported in a Spanish study, gain-framed messages i.e. emphasizing the benefits of behavioral change, can increase condom use [35]. Consequently, focusing on the social benefits of protected sex could be a more effective way to address the issue for the youth community.

It is important to highlight that the youth in our study also expressed a *need* for visiting the YHC repeatedly as they continued to expose themselves to risk of contracting STI although they were aware of the possible long term medical consequences of *C.trachomatis* i.e. infertility. This finding is in line with other studies reporting that STI knowledge does not translate into increased condom use [36, 37].

### Health beliefs and re-testing for C.Trachomatis among the youth

Health beliefs influence the need for and utilization of health services [20]. In our study we found that two specific health beliefs: *“C.trachomatis* is a quick fix and *C.trachomatis* is not a serious infection”, promote both risky sexual behavior and the need for repeated testing.

The conceptualization of *C.trachomatis* as a mild and easily treatable disease might explain why young people in Sweden express moderate concerns about STI diagnosis and perceive themselves at little risk of contracting STI [38, 39]. In addition, it might explain why we found that neither a positive nor a negative *C.trachomatis* diagnosis led to long term preventive behavioural change. Previous studies have indicated similar trends. For example, one US study showed that condom use improved after a positive STI test but reverted after 3 months [40].

Our findings also highlight that, for our informants, testing negative for *C.trachomatis* provided a false sense of security that validated their choice of sexual partners and their unsafe sexual behavior. This is in line with previous studies reporting that youth who tested negative for STI tended to increase the number of sexual partners, the number of unprotected intercourses [12] and were less likely to use a condom 1 year after testing [13]. Clearly, as also described elsewhere [13], a negative result test seemed to inhibit the participants’ behavioral change.

*Health beliefs* about *C.trachomatis* are thus important to understand in relation to the success of the screening program and primary preventive strategies. According to our finding expanding existing testing services alone might not make youth adopt safer sexual behaviour. The “quick fix” notion might in fact have the opposite effect on sexual risk taking. Challenging this quick fix notion could be important to promote preventive sexual behavior.

### The environment – The Swedish health care system

The process of repeatedly testing for *C.trachomatis* was not an isolated process driven just by the individual but was also facilitated by the characteristics of the Swedish health care system. Youth have access to a system that assures free testing, free treatment and easy geographical access to health services [26]. While the YHC is a well-established part of Swedish health care, access to youth-friendly services for sexual and reproductive healthcare varies widely between countries [41, 42]. Evaluation and comparison of the effect of youth-friendly clinics on health outcomes such as teenage pregnancy and STI prevalence are thus difficult to assess on a global level, furthermore reporting of the above events are often inadequate [42]. However, the Swedish approach of easy access to the YHC, while commendable should be critically evaluated in terms of its focus on preventive work with regard to STIs. STI prevalence in the youth population might indicate that more can be done in this regard.

### Re-testing: A “safe approach” to “unsafe sex”

Our analysis showed that the interaction between the aforementioned factors has translated into specific health behaviour. Youth in our study seemed to have found a “safe approach” to “unsafe sex”. Testing was looked upon as a normal procedure after unsafe sex, a substitute to using condoms. We have not found other studies that report similar findings in this context. It can of course be argued that testing after unprotected sex is better than not testing after unprotected sex. The positive side of this behaviour is that infections are found and treated, partners are notified, and the transmission chain is interrupted. While this is not disputed, testing repeatedly for *C.trachomatis* allowed youth in our study to continue their sexual risk-taking. As described elsewhere, in the context of HIV prevention, repeat testing allows continued high-risk sexual behavior by confirming that past and current behaviors are safe [43]. Findings from our study indicate similar relationship between repeat testing and sexual risk-taking. This stresses the need for focusing on primary preventive strategies. Furthermore it is clear that young people must be involved in the solutions to halter the epidemic of *C.trachomatis* in Sweden and elsewhere.

### Limitations

One limitation includes the method used; in-depth interviews on a sensitive subject could prevent participants from answering truthfully. The interviewer is also a clinic based midwife and it is possible that youth felt intimidated by this or even forced to answer the questions posed during the interview in a particular way. However, we used several strategies to avoid these pitfalls - anonymity was repeatedly assured, the interviews were held in homelike private rooms and the interviewer were dressed in everyday casual clothes, not in the work uniform. To avoid preconceptions and assumptions that could affect the interpretation of the data, each step in the analysis process was done by two researchers with different background. By continuously reflecting on the relationship to the respondents and the subject itself, misinterpretation of the data was thought to be avoided. Trustworthiness of the data was amplified during the interview by the interviewer summarizing the responses to the participant who could confirm or not confirm the accuracy. Furthermore the findings in the present study were triangulated by findings in a subsequent, yet unpublished study [44].

## Conclusions

Our study showed that testing repeatedly for *C.trachomatis* is a process driven by the interaction between the young peoples´ health beliefs, their conceptualization of re-testing as a replacement for condom use and their the fear of negative social consequences of infecting a close peer.

Testing services are key elements needed for *C.trachomatis* prevention. However, they must not be perceived as a replacement for safe sex. Testing services must therefore be complemented with a much stronger emphasis on safe sex, especially for those who attend the clinic repeatedly. Otherwise, the easily accessible testing services risk counteracting their purpose.

Youth perceptions and beliefs about *C.trachomatis* can be used as triggers for behavioural changes. Health interventions targeted to the risk group of repeat testers should appeal to their motivation to reduce anxiety related to unprotected sex i.e. avoiding possible social stigma. These should guide the development and evaluation of appropriate interventions to increase condom use.

## Additional file

Additional file 1: Interview guide Youths. (DOC 31 kb)

## Abbreviations

*C.trachomatis*: Chlamydia trachomatis
HIV: Human Immunodeficiency Virus
HPV: Human Papilloma Virus
STI: Sexually Transmitted Infection
YHC: Youth Health Clinic
