# Supplementary material for: Repeat testing for chlamydia trachomatis, a “safe approach” to unsafe sex? a qualitative exploration among youth in Stockholm
Source: BMC Health Serv Res. 2017 Nov 15;17:730. doi: 10.1186/s12913-017-2681-6 (PMC5688721; doi:10.1186/s12913-017-2681-6)
Supplement: Additional file 1: — Interview guide Youths. (DOC 31 kb) [file 12913_2017_2681_MOESM1_ESM.doc]

**Interview guide Youths**

| **Aspects to explore** | **Questions** |
| --- | --- |
| Introductory questions | Tell me a little bit about yourself.  Explore age, school, work, and family. |
| Using the clinic | Tell me about your experiences of visiting the clinic.  What is the most common reason you come here? |
| Testing for Chlamydia | Can you tell me about your experiences being tested for Chlamydia?  What do you think about / know about Chlamydia |
| Sexual risk-taking | What do you consider being a risky sexual behaviour?  What do you consider having a safe sexual life is?  Alcohol?  Multiple partners?  Emotional risk? |
| Aspects influencing repeated testing | Can you tell me a little bit more about being tested repeatedly for Chlamydia?  What do you think influences young people to test repeatedly for Chlamydia?  What happens when you get a test?  What about condom use and repeated testing?  What about other contraceptives and repeated testing?  Trust in partner? What do you think about that?  Does the fact that testing is free matter?  Security for HIV  Security for Chlamydia  What would happen if people would start to pay for it? |
| Security in repeated testing | Tell me about how you feel when the test result is negative?  How does the negative test result influence your?  If you would test positive for Chlamydia how would that make you feel?  Would a positive result influence you in any way? |
